# Supplementary material for: Functional ability and quality of life in critical illness survivors with intensive care unit acquired weakness: A secondary analysis of a randomised controlled trial
Source: PLoS One. 2020 Mar 4;15(3):e0229725. doi: 10.1371/journal.pone.0229725 (PMC7056321; doi:10.1371/journal.pone.0229725)
Supplement: S1 Table — (PDF) [file pone.0229725.s001.pdf]

S1 Table. Timetable for primary and secondary outcome measures.

	ICU discharge	hospital discharge	6 months follow-up
Medical Research Council sum-score (MRC-SS)	x		
Functional Independence Measure (FIM)	x	x	
6-Minute Walk Test (6MWT)		x	
Timed “Up & Go” Test (TUG)		x	
Hospital length of stay, discharge destination, ICU readmissions		x	
Mortality		x	x
Short Form 36 (SF-36)			x
