# Supplementary material for: Functional ability and quality of life in critical illness survivors with intensive care unit acquired weakness: A secondary analysis of a randomised controlled trial
Source: PLoS One. 2020 Mar 4;15(3):e0229725. doi: 10.1371/journal.pone.0229725 (PMC7056321; doi:10.1371/journal.pone.0229725)
Supplement: S2 Table — (PDF) [file pone.0229725.s002.pdf]

S1 File.

S2 Table. Baseline characteristics based upon completed and missing MRC-SS.

variable	n	completed MRC-SS	n	missing MRC-SS	p-value
**MRC sum-score at ICU discharge (0-60)**	83	45 [38.5, 54]		NA [NA, NA]	
**Randomized to non-standard intervention**	83	40 (48%)	32	18 (56%)	**0.57**
**Age (years)**	83	67.5 [55.55, 75.4]	32	67.6 [57.7, 73.53]	**0.84**
**Gender (male)**	83	52 (63%)	32	25 (78%)	**0.17**
**BMI (kg/m^2)**	83	26.2 [23.6, 31.35]	32	26.35 [24.62, 28.1]	**0.72**
**Weight (kg)**	83	80 [66, 90]	32	82 [74.75, 90.25]	**0.35**
**ICU diagnosis on ICU admission**	83		32		**0.57**
gastroenterology		11 (13%)		3 (9%)	
heart surgery		18 (22%)		3 (9%)	
hemodynamic insufficiency		16 (19%)		10 (31%)	
neurology / neurosurgery		4 (5%)		5 (16%)	
other		1 (1%)		1 (3%)	
other surgery		11 (13%)		3 (9%)	
respiratory insufficiency		20 (24%)		5 (16%)	
trauma		2 (2%)		2 (6%)	
**APACHE II score (0-71) [a]**	83	21 [17, 26]	32	27 [21.5, 30]	**< 0.001**
**SOFA score (0-24) [b]**	83	8 [6, 10]	32	10 [8, 14.25]	**< 0.001**
**ICU days until study inclusion**	83	1.71 [0.85, 2.57]	32	1.82 [1.08, 2.39]	**0.60**
**Restricted in activities of daily living (ADL)**	80	8 (10%)	31	5 (16%)	**0.57**
**NYHA symptoms (stage 2 to 4)**	80	36 (45%)	31	12 (39%)	**0.70**
**Dyspnoea symptoms**	80	20 (25%)	31	10 (32%)	**0.59**
**Hematologic malignancy**	80	3 (4%)	31	2 (6%)	**0.92**
**Immunosuppression**	80	11 (14%)	31	5 (16%)	**0.98**
**Liver disease**	80	7 (9%)	31	8 (26%)	**0.04**
**Chronic dialysis**	80	0 (0%)	31	0 (0%)	

[a] at ICU admission

[b] at study inclusion

Comparison of baseline characteristics of patients with observed MRC-SS and patients for whom we could not collect MRC-SS. Data are presented as median [25%, 75%] or frequencies (%).

Abbreviations: NA = not available, NYHA = New York Heart Association, BMI = Body Mass Index, APACHE = Acute Physiology and Chronic Health Evaluation, SOFA = Sequential Organ Failure Assessment
