# Supplementary material for: Functional ability and quality of life in critical illness survivors with intensive care unit acquired weakness: A secondary analysis of a randomised controlled trial
Source: PLoS One. 2020 Mar 4;15(3):e0229725. doi: 10.1371/journal.pone.0229725 (PMC7056321; doi:10.1371/journal.pone.0229725)
Supplement: S3 Table — (PDF) [file pone.0229725.s003.pdf]

S3 Table. Baseline characteristics for subjects with missing MRC-SS values based on reason for missingness.

variable	n	died	n	unable to follow commands	n	other
**MRC sum-score at ICU discharge (0-60)**	0	NA [NA, NA]	0	NA [NA, NA]	0	NA [NA, NA]
**Randomized to non-standard intervention**	16	9 (56%)	11	6 (55%)	5	3 (60%)
**Age (years)**	16	67.6 [60.5, 71.7]	11	63.5 [52.25, 73.75]	5	75.2 [73, 76.5]
**Gender male**	16	12 (75%)	11	9 (82%)	5	4 (80%)
**BMI (kg/m^2)**	16	26.35 [24.8, 27.7]	11	28.4 [25.4, 30.1]	5	24.7 [23.4, 24.7]
**Weight (kg)**	16	84.5 [73, 90.75]	11	90 [80, 93]	5	80 [60, 80]
**ICU diagnosis on ICU admission**	16		11		5	
gastroenterology		3 (19%)		0 (0%)		0 (0%)
heart surgery		1 (6%)		2 (18%)		0 (0%)
hemodynamic insufficiency		5 (31%)		2 (18%)		3 (60%)
neurology / neurosurgery		0 (0%)		5 (45%)		0 (0%)
other		1 (6%)		0 (0%)		0 (0%)
other surgery		2 (12%)		1 (9%)		0 (0%)
respiratory insufficiency		3 (19%)		0 (0%)		2 (40%)
trauma		1 (6%)		1 (9%)		0 (0%)
**APACHE II score [a] (0-71)**	16	28 [25, 31]	11	24 [17, 29.5]	5	24 [23, 30]
**SOFA score (0-24) [b]**	16	13 [9.75, 15]	11	8 [7, 10]	5	7 [6, 11]
**ICU days until study inclusion**	16	1.85 [0.95, 2.61]	11	1.77 [1.59, 2.15]	5	1.1 [1.01, 2.35]
**Restricted in activities of daily living**	15	4 (27%)	11	1 (9%)	5	0 (0%)
**NYHA symptoms (stage 2 to 4)**	15	7 (47%)	11	2 (18%)	5	3 (60%)
**Dyspnoea symptoms**	15	5 (33%)	11	1 (9%)	5	4 (80%)
**Hematologic malignancy**	15	2 (13%)	11	0 (0%)	5	0 (0%)
**Immunosuppression**	15	3 (20%)	11	1 (9%)	5	1 (20%)
**Liver disease**	15	4 (27%)	11	3 (27%)	5	1 (20%)
**Chronic dialysis**	15	0 (0%)	11	0 (0%)	5	0 (0%)

[a] at ICU admission

[b] at study inclusion

Further interpretation of differences between observed and missing MRC-SS with reference to the three reasons why MRC-SS was not collected. APACHE II and SOFA scores were higher in participants whose MRC-SS could not be assessed because of ICU death, whereas liver disease frequency was equal in unassessed patients who ‘died’ or were ‘unable to follow commands’. Data are presented as median [25%, 75%] or frequencies (%).

Abbreviations: NA = not available, NYHA = New York Heart Association, BMI = Body Mass Index, APACHE = Acute Physiology and Chronic Health Evaluation, SOFA = Sequential Organ Failure Assessment
