# Supplementary material for: Functional ability and quality of life in critical illness survivors with intensive care unit acquired weakness: A secondary analysis of a randomised controlled trial
Source: PLoS One. 2020 Mar 4;15(3):e0229725. doi: 10.1371/journal.pone.0229725 (PMC7056321; doi:10.1371/journal.pone.0229725)
Supplement: S4 Table — This includes German norm-based (1994) standardized sum-scores (T-values) for SF-36. (PDF) [file pone.0229725.s004.pdf]

S4 Table. Sensitivity analysis comparing ICUAW versus non-ICUAW. This includes German norm-based (1994) standardized sum-scores (T-values) for SF-36.

Variable	n	ICUAW	n	non-ICUAW	p-value
**Primary outcomes at hospital discharge**					
6MWT (m)	45	165 [80 - 260]	28	222.5 [128 - 382.5]	0.017
FIM (18-126)	44	105.5 [88.5 - 117.5]	29	112 [97 - 123]	0.028
**Secondary hospital outcomes**					
Timed ‘Up & Go ‘test (s) at hospital discharge	35	22.5 [14 - 32]	22	14 [8 - 24]	0.028
Hospital length of stay after ICU discharge (days)	49	18.82 [13.82 - 30.28]	34	11.16 [7.01 - 20.31]	0.005
**SF-36: quality of life after 6 months** (based on US-population 1990)					
Physical health (sum-score)	25	42.92 [32.74 - 48.23]	24	42.18 [36.35 - 49.30]	0.904
Mental health (sum-score)	25	48.46 [44.40 - 55.75]	24	51.86 [45.33 - 56.20]	0.496
**SF-36: quality of life after 6 months** (based on German-population 1994)					
Physical health (sum-score)	25	42.83 [32.48 - 48.91]	24	42.32 [37.50 - 49.61]	0.826
Mental health (sum-score)	25	45.37 [41.69 - 55.00]	24	48.55 [41.90 - 55.60]	0.447

Data are presented as median [IQR: 25% - 75%] or frequencies (%). This sensitivity analysis compares the data of participants without ICUAW to the pooled data of participants with severe and moderate ICUAW with the non-parametric Mann-Whitney-U tests for between-group comparisons.
