# Supplementary material for: Functional ability and quality of life in critical illness survivors with intensive care unit acquired weakness: A secondary analysis of a randomised controlled trial
Source: PLoS One. 2020 Mar 4;15(3):e0229725. doi: 10.1371/journal.pone.0229725 (PMC7056321; doi:10.1371/journal.pone.0229725)
Supplement: S6 Table — (PDF) [file pone.0229725.s006.pdf]

S6 Table. Sensitivity analysis for the regression model either with or without the randomisation group or with or without the ADL variable.

	n	Adjusted effect without randomisation group (95% CI)	p-value	n	Adjusted effect without ADL (95% CI)	p-value
female		*Reference*			*Reference*	
**male**	80	5.62 (0.86 to 10.39)	0.024	83	5.62 (0.71 to 10.52)	0.028
control group		-			*Reference*	
**experimental group**	80	-		83	-0.16 (-5.06 to 4.75)	0.950
**SOFA score**	80	-0.53 (-1.24 to 0.18)	0.145	83	-0.42 (-1.14 to 0.30)	0.254
**Length of ICU stay at original hospital (days)**	80	-0.26 (-0.53 to 0.01)	0.059	83	-0.28 (-0.55 to -0.01)	0.049
Not restricted in ADL		*Reference*			-	
**Restricted in ADL**	80	-6.15 (-13.89 to 1.59)	0.124	83	-	
Mobilisation level in ICU: out-of-bed		*Reference*			*Reference*	
**Mobilisation level in ICU: in-bed**	80	-24.52 (-36.90 to -12.15)	<0.001	83	-24.30 (-36.99 to -11.60)	<0.001
**Mobilisation level in ICU: edge-of-bed**	80	-7.41 (-12.74 to -2.08)	0.008	83	-8.25 (-13.74 to -2.76)	0.004

Significance of the chosen variables in a regression model with MRC-SS as response. Adjusted regression includes all listed explanatory variables either with or without the randomisation group and with or without the ADL (Activities of Daily Living) variable. The robust estimations yield the same results with non-significant bias' tests (with the null: presence of the bias due to lack of robustness or outlying observations). None of the terms revealed non-linearity when fitting a regression model allowing non-linear dependence (via penalized splines) on explanatory variables.

Abbreviations: SOFA = Sequential Organ Failure Assessment, ADL = activities of daily living
