# Supplementary material for: Functional ability and quality of life in critical illness survivors with intensive care unit acquired weakness: A secondary analysis of a randomised controlled trial
Source: PLoS One. 2020 Mar 4;15(3):e0229725. doi: 10.1371/journal.pone.0229725 (PMC7056321; doi:10.1371/journal.pone.0229725)
Supplement: S1 File — (PDF) [file pone.0229725.s007.pdf]

All regressions' outputs for Eggmann et al

January 31, 2020

Crude regression models for MRC-SS

Crude model for sex

```
##
## Call:
## lm(formula = MRC_SS_last ~ sex, data = icuaw)
##
## Residuals:
##     Min      1Q  Median      3Q     Max
## -40.038  -5.568   1.962   8.932  16.903
##
## Coefficients:
##             Estimate Std. Error t value Pr(>|t|)
## (Intercept)   39.097      2.153  18.160   <2e-16 ***
## sexmale        6.942      2.720   2.552   0.0126 *
## ---
## Signif. codes:  0 '***' 0.001 '**' 0.01 '*' 0.05 '.' 0.1 ' ' 1
##
## Residual standard error: 11.99 on 81 degrees of freedom
##   (32 observations deleted due to missingness)
## Multiple R-squared:  0.07443,    Adjusted R-squared:  0.063
## F-statistic: 6.513 on 1 and 81 DF,  p-value: 0.01259
```

Crude model for SOFA

```
##
## Call:
## lm(formula = MRC_SS_last ~ sofa_score, data = icuaw)
##
## Residuals:
##     Min      1Q  Median      3Q     Max
## -38.247  -4.480   1.753   9.221  20.818
##
## Coefficients:
##             Estimate Std. Error t value Pr(>|t|)
## (Intercept)  48.6785     3.3704  14.443   <2e-16 ***
## sofa_score   -0.6331     0.3740  -1.693   0.0943 .
## ---
## Signif. codes:  0 '***' 0.001 '**' 0.01 '*' 0.05 '.' 0.1 ' ' 1
##
## Residual standard error: 12.24 on 81 degrees of freedom
##   (32 observations deleted due to missingness)
## Multiple R-squared:  0.03418,    Adjusted R-squared:  0.02225
## F-statistic: 2.866 on 1 and 81 DF,  p-value: 0.09429
```

Crude model for hospital length of stay (LOS)

```
##
## Call:
## lm(formula = MRC_SS_last ~ LOS_ICU_Insel_W, data = icuaw)
##
## Residuals:
##     Min      1Q  Median      3Q     Max
## -38.416  -5.086   1.929  10.053  22.105
##
## Coefficients:
##                 Estimate Std. Error t value Pr(>|t|)
## (Intercept)      45.4135     1.8535  24.502   <2e-16 ***
## LOS_ICU_Insel_W  -0.2124     0.1373  -1.547    0.126
## ---
## Signif. codes:  0 '***' 0.001 '**' 0.01 '*' 0.05 '.' 0.1 ' ' 1
##
## Residual standard error: 12.28 on 81 degrees of freedom
##   (32 observations deleted due to missingness)
## Multiple R-squared:  0.02868,    Adjusted R-squared:  0.01669
## F-statistic: 2.392 on 1 and 81 DF,  p-value: 0.1259
```

Crude model for ADL (Activities of Daily Living)

```
##
## Call:
## lm(formula = MRC_SS_last ~ ADL_restr, data = icuaw)
##
## Residuals:
##     Min      1Q  Median      3Q     Max
## -37.875  -4.875   1.125  10.125  21.500
##
## Coefficients:
##              Estimate Std. Error t value Pr(>|t|)
## (Intercept)    43.875      1.444  30.377   <2e-16 ***
## ADL_restryes   -5.375      4.567  -1.177    0.243
## ---
## Signif. codes:  0 '***' 0.001 '**' 0.01 '*' 0.05 '.' 0.1 ' ' 1
##
## Residual standard error: 12.26 on 78 degrees of freedom
##   (35 observations deleted due to missingness)
## Multiple R-squared:  0.01745,    Adjusted R-squared:  0.004848
## F-statistic: 1.385 on 1 and 78 DF,  p-value: 0.2429
```

Crude model for mobilization level during the ICU stay (bed_eob)

```
##
## Call:
## lm(formula = MRC_SS_last ~ Bed_EOB_OOB, data = icuaw)
##
## Residuals:
##     Min      1Q  Median      3Q     Max
```

```
## -33.577  -5.520  -0.463   9.037  20.423
##
## Coefficients:
##              Estimate Std. Error t value Pr(>|t|)
## (Intercept)    46.463      1.551  29.948  < 2e-16 ***
## Bed_EOB_OOB1  -23.796      6.763  -3.519 0.000718 ***
## Bed_EOB_OOB2   -6.886      2.721  -2.530 0.013359 *
## ---
## Signif. codes:  0 '***' 0.001 '**' 0.01 '*' 0.05 '.' 0.1 ' ' 1
##
## Residual standard error: 11.4 on 80 degrees of freedom
##   (32 observations deleted due to missingness)
## Multiple R-squared:  0.1731, Adjusted R-squared:  0.1524
## F-statistic: 8.371 on 2 and 80 DF,  p-value: 0.0005001
```

Crude model for randomization group

```
##
## Call:
## lm(formula = MRC_SS_last ~ rand_group, data = icuaw)
##
## Residuals:
##     Min      1Q  Median      3Q     Max
## -38.419  -5.909   1.581  10.081  17.600
##
## Coefficients:
##               Estimate Std. Error t value Pr(>|t|)
## (Intercept)     44.419      1.894   23.46   <2e-16 ***
## rand_groupyes   -2.019      2.728   -0.74    0.461
## ---
## Signif. codes:  0 '***' 0.001 '**' 0.01 '*' 0.05 '.' 0.1 ' ' 1
##
## Residual standard error: 12.42 on 81 degrees of freedom
##   (32 observations deleted due to missingness)
## Multiple R-squared:  0.006715,   Adjusted R-squared:  -0.005548
## F-statistic: 0.5476 on 1 and 81 DF,  p-value: 0.4614
```

Adjusted regression models

Adjusted model with randomization

```
##
## Call:
## lm(formula = MRC_SS_last ~ rand_group + sex + sofa_score + LOS_ICU_Insel_W +
##     ADL_restr + Bed_EOB_OOB, data = icuaw)
##
## Residuals:
##     Min      1Q  Median      3Q     Max
## -38.161  -4.930  -0.029   5.820  19.419
##
## Coefficients:
##                 Estimate Std. Error t value Pr(>|t|)
```

```
## (Intercept)      50.9038     3.7402  13.610  < 2e-16 ***
## rand_groupyes    -0.6588     2.5185  -0.262 0.794377
## sexmale           5.5125     2.4837   2.220 0.029603 *
## sofa_score       -0.5481     0.3682  -1.488 0.141012
## LOS_ICU_Insel_W  -0.2595     0.1386  -1.873 0.065159 .
## ADL_restryes     -6.1797     3.9748  -1.555 0.124400
## Bed_EOB_OOB1    -24.5729     6.3580  -3.865 0.000241 ***
## Bed_EOB_OOB2     -7.2011     2.8480  -2.528 0.013648 *
## ---
## Signif. codes:  0 '***' 0.001 '**' 0.01 '*' 0.05 '.' 0.1 ' ' 1
##
## Residual standard error: 10.48 on 72 degrees of freedom
##   (35 observations deleted due to missingness)
## Multiple R-squared:  0.3367, Adjusted R-squared:  0.2722
## F-statistic: 5.221 on 7 and 72 DF,  p-value: 7.564e-05
```

Adjusted model with randomization without ADL (activities of daily living)

```
##
## Call:
## lm(formula = MRC_SS_last ~ rand_group + sex + sofa_score + LOS_ICU_Insel_W +
##     Bed_EOB_OOB, data = icuaw)
##
## Residuals:
##     Min      1Q  Median      3Q     Max
## -36.640  -5.510   0.522   5.943  20.145
##
## Coefficients:
##                 Estimate Std. Error t value Pr(>|t|)
## (Intercept)      49.5384     3.7096  13.354  < 2e-16 ***
## rand_groupyes    -0.1580     2.5032  -0.063 0.949834
## sexmale           5.6153     2.5020   2.244 0.027721 *
## sofa_score       -0.4218     0.3667  -1.150 0.253621
## LOS_ICU_Insel_W  -0.2790     0.1394  -2.002 0.048874 *
## Bed_EOB_OOB1    -24.2951     6.4789  -3.750 0.000343 ***
## Bed_EOB_OOB2     -8.2512     2.7996  -2.947 0.004255 **
## ---
## Signif. codes:  0 '***' 0.001 '**' 0.01 '*' 0.05 '.' 0.1 ' ' 1
##
## Residual standard error: 10.7 on 76 degrees of freedom
##   (32 observations deleted due to missingness)
## Multiple R-squared:  0.3076, Adjusted R-squared:  0.253
## F-statistic: 5.628 on 6 and 76 DF,  p-value: 7.093e-05
```

Robust adjusted model with randomization with ADL

```
##
## Call:
## lmRob(formula = MRC_SS_last ~ rand_group + sex + sofa_score +
##     LOS_ICU_Insel_W + ADL_restr + Bed_EOB_OOB, data = icuaw)
##
## Residuals:
##     Min      1Q  Median      3Q     Max
```

```
## -42.418  -5.870  -0.301   5.927  17.553
##
## Coefficients:
##                 Estimate Std. Error t value Pr(>|t|)
## (Intercept)      51.4610     4.1988  12.256  < 2e-16 ***
## rand_groupyes    -1.5512     2.8752  -0.540 0.591196
## sexmale           6.2636     2.8193   2.222 0.029449 *
## sofa_score       -0.6341     0.4198  -1.511 0.135247
## LOS_ICU_Insel_W  -0.2410     0.1721  -1.401 0.165617
## ADL_restryes     -8.0101     4.4582  -1.797 0.076573 .
## Bed_EOB_OOB1    -24.5001     7.1102  -3.446 0.000954 ***
## Bed_EOB_OOB2     -3.7359     3.3074  -1.130 0.262416
## ---
## Signif. codes:  0 '***' 0.001 '**' 0.01 '*' 0.05 '.' 0.1 ' ' 1
##
## Residual standard error: 9.451 on 72 degrees of freedom
## Multiple R-Squared: 0.2855
##
## Test for Bias:
##             statistic p-value
## M-estimate      1.055  0.9979
## LS-estimate     6.355  0.6075
## 35 observations deleted due to missingness
```

Adjusted model without randomization with ADL

```
##
## Call:
## lm(formula = MRC_SS_last ~ sex + sofa_score + LOS_ICU_Insel_W +
##     ADL_restr + Bed_EOB_OOB, data = icuaw)
##
## Residuals:
##     Min      1Q  Median      3Q     Max
## -37.747  -4.767   0.264   5.786  19.234
##
## Coefficients:
##                 Estimate Std. Error t value Pr(>|t|)
## (Intercept)      50.4916     3.3703  14.981  < 2e-16 ***
## sexmale           5.6227     2.4320   2.312 0.023601 *
## sofa_score       -0.5324     0.3610  -1.475 0.144564
## LOS_ICU_Insel_W  -0.2628     0.1371  -1.917 0.059201 .
## ADL_restryes     -6.1510     3.9479  -1.558 0.123546
## Bed_EOB_OOB1    -24.5234     6.3145  -3.884 0.000224 ***
## Bed_EOB_OOB2     -7.4073     2.7192  -2.724 0.008063 **
## ---
## Signif. codes:  0 '***' 0.001 '**' 0.01 '*' 0.05 '.' 0.1 ' ' 1
##
## Residual standard error: 10.41 on 73 degrees of freedom
##   (35 observations deleted due to missingness)
## Multiple R-squared:  0.3361, Adjusted R-squared:  0.2815
## F-statistic: 6.158 on 6 and 73 DF,  p-value: 2.915e-05
```

Robust adjusted model without randomization with ADL

```
##
## Call:
## lmRob(formula = MRC_SS_last ~ sex + sofa_score + LOS_ICU_Insel_W +
##     ADL_restr + Bed_EOB_OOB, data = icuaw)
##
## Residuals:
##      Min       1Q   Median       3Q      Max
## -41.7261  -5.6808  -0.6937   5.5782  18.2447
##
## Coefficients:
##                 Estimate Std. Error t value Pr(>|t|)
## (Intercept)      50.6050     3.6188  13.984  < 2e-16 ***
## sexmale           6.3926     2.6308   2.430  0.01756 *
## sofa_score       -0.5942     0.3868  -1.536  0.12875
## LOS_ICU_Insel_W  -0.2484     0.1474  -1.685  0.09624 .
## ADL_restryes     -8.1376     4.2336  -1.922  0.05849 .
## Bed_EOB_OOB1    -24.4793     6.7518  -3.626  0.00053 ***
## Bed_EOB_OOB2     -3.9459     2.9778  -1.325  0.18927
## ---
## Signif. codes:  0 '***' 0.001 '**' 0.01 '*' 0.05 '.' 0.1 ' ' 1
##
## Residual standard error: 9.439 on 73 degrees of freedom
## Multiple R-Squared: 0.2815
##
## Test for Bias:
##             statistic p-value
## M-estimate   -0.06088   1.000
## LS-estimate   6.60663   0.471
## 35 observations deleted due to missingness
```

Additive adjusted model without randomization with ADL

```
##
## Family: gaussian
## Link function: identity
##
## Formula:
## MRC_SS_last ~ sex + sofa_score + s(LOS_ICU_Insel_W) + ADL_restr +
##     Bed_EOB_OOB
##
## Parametric coefficients:
##              Estimate Std. Error t value Pr(>|t|)
## (Intercept)   48.1054     3.4461  13.959  < 2e-16 ***
## sexmale        5.4805     2.4450   2.242 0.028043 *
## sofa_score    -0.5280     0.3605  -1.465 0.147346
## ADL_restryes  -6.1015     3.9419  -1.548 0.125993
## Bed_EOB_OOB1 -24.7749     6.3227  -3.918 0.000199 ***
## Bed_EOB_OOB2  -7.5412     2.7276  -2.765 0.007213 **
## ---
## Signif. codes:  0 '***' 0.001 '**' 0.01 '*' 0.05 '.' 0.1 ' ' 1
##
```

```
## Approximate significance of smooth terms:
##                      edf Ref.df     F p-value
## s(LOS_ICU_Insel_W) 1.225  1.414 2.344   0.091 .
## ---
## Signif. codes:  0 '***' 0.001 '**' 0.01 '*' 0.05 '.' 0.1 ' ' 1
##
## R-sq.(adj) =  0.284   Deviance explained = 34.1%
## GCV = 118.77  Scale est. = 108.05    n = 80
```

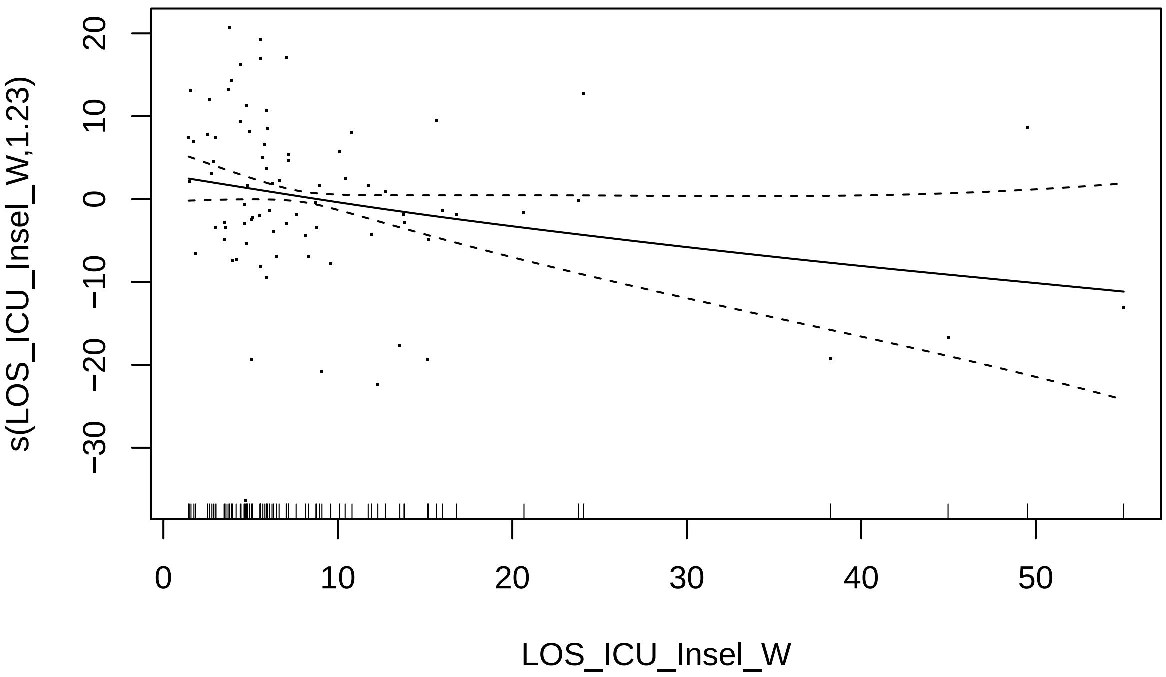

The edf is very close to 1 (1.4) and the plot shows rather a linear term, so the non-linear model is not worth using

Crude regression models for 6MWT

Crude model for sex

```
##
## Call:
## lm(formula = six_MWT_effect ~ sex, data = icuaw)
##
## Residuals:
##     Min      1Q  Median      3Q     Max
## -235.10 -125.10  -26.94  121.22  414.90
##
## Coefficients:
##             Estimate Std. Error t value Pr(>|t|)
```

```
## (Intercept)   178.78      27.43   6.519 5.41e-09 ***
## sexmale        56.31      34.86   1.616     0.11
## ---
## Signif. codes:  0 '***' 0.001 '**' 0.01 '*' 0.05 '.' 0.1 ' ' 1
##
## Residual standard error: 155.1 on 82 degrees of freedom
##   (31 observations deleted due to missingness)
## Multiple R-squared:  0.03085,    Adjusted R-squared:  0.01903
## F-statistic:  2.61 on 1 and 82 DF,  p-value: 0.11
```

Crude model for SOFA

```
##
## Call:
## lm(formula = six_MWT_effect ~ sofa_score, data = icuaw)
##
## Residuals:
##     Min      1Q  Median      3Q     Max
## -226.06 -121.12  -17.66  100.24  443.97
##
## Coefficients:
##             Estimate Std. Error t value Pr(>|t|)
## (Intercept)  284.474     43.204   6.584 4.05e-09 ***
## sofa_score    -8.345      4.686  -1.781   0.0786 .
## ---
## Signif. codes:  0 '***' 0.001 '**' 0.01 '*' 0.05 '.' 0.1 ' ' 1
##
## Residual standard error: 154.6 on 82 degrees of freedom
##   (31 observations deleted due to missingness)
## Multiple R-squared:  0.03724,    Adjusted R-squared:  0.02549
## F-statistic: 3.171 on 1 and 82 DF,  p-value: 0.07864
```

Crude model for hospital length of stay (LOS)

```
##
## Call:
## lm(formula = six_MWT_effect ~ LOS_ICU_Insel_W, data = icuaw)
##
## Residuals:
##     Min      1Q  Median      3Q     Max
## -210.75 -130.91  -26.51  100.86  429.18
##
## Coefficients:
##                 Estimate Std. Error t value Pr(>|t|)
## (Intercept)      231.005     23.885   9.672 3.33e-15 ***
## LOS_ICU_Insel_W   -1.842      1.771  -1.040    0.301
## ---
## Signif. codes:  0 '***' 0.001 '**' 0.01 '*' 0.05 '.' 0.1 ' ' 1
##
## Residual standard error: 156.6 on 82 degrees of freedom
##   (31 observations deleted due to missingness)
## Multiple R-squared:  0.01302,    Adjusted R-squared:  0.000984
## F-statistic: 1.082 on 1 and 82 DF,  p-value: 0.3014
```

Crude model for ADL (Activities of Daily Living)

```
##
## Call:
## lm(formula = six_MWT_effect ~ ADL_restr, data = icuaw)
##
## Residuals:
##     Min      1Q  Median      3Q     Max
## -220.76 -129.51  -15.76   99.24  429.24
##
## Coefficients:
##             Estimate Std. Error t value Pr(>|t|)
## (Intercept)   220.76      18.22  12.117   <2e-16 ***
## ADL_restryes  -49.47      62.36  -0.793     0.43
## ---
## Signif. codes:  0 '***' 0.001 '**' 0.01 '*' 0.05 '.' 0.1 ' ' 1
##
## Residual standard error: 157.8 on 80 degrees of freedom
##   (33 observations deleted due to missingness)
## Multiple R-squared:  0.007807,   Adjusted R-squared:  -0.004595
## F-statistic: 0.6295 on 1 and 80 DF,  p-value: 0.4299
```

Crude model for mobilization level during the ICU stay (bed_eob)

```
##
## Call:
## lm(formula = six_MWT_effect ~ Bed_EOB_OOB, data = icuaw)
##
## Residuals:
##     Min      1Q  Median      3Q     Max
## -234.71 -117.16  -25.51  106.59  415.29
##
## Coefficients:
##             Estimate Std. Error t value Pr(>|t|)
## (Intercept)   234.71      22.39  10.481   <2e-16 ***
## Bed_EOB_OOB1 -114.42      62.77  -1.823    0.072 .
## Bed_EOB_OOB2  -33.40      36.49  -0.915    0.363
## ---
## Signif. codes:  0 '***' 0.001 '**' 0.01 '*' 0.05 '.' 0.1 ' ' 1
##
## Residual standard error: 155.2 on 81 degrees of freedom
##   (31 observations deleted due to missingness)
## Multiple R-squared:  0.04258,    Adjusted R-squared:  0.01894
## F-statistic: 1.801 on 2 and 81 DF,  p-value: 0.1716
```

Crude model for randomization group

```
##
## Call:
## lm(formula = six_MWT_effect ~ rand_group, data = icuaw)
##
## Residuals:
```

```
##      Min       1Q  Median      3Q     Max
## -223.38 -124.80  -24.09  103.95  426.63
##
## Coefficients:
##               Estimate Std. Error t value Pr(>|t|)
## (Intercept)     223.38      24.87   8.980 7.87e-14 ***
## rand_groupyes   -18.58      34.37  -0.541     0.59
## ---
## Signif. codes:  0 '***' 0.001 '**' 0.01 '*' 0.05 '.' 0.1 ' ' 1
##
## Residual standard error: 157.3 on 82 degrees of freedom
##   (31 observations deleted due to missingness)
## Multiple R-squared:  0.003552,   Adjusted R-squared:  -0.0086
## F-statistic: 0.2923 on 1 and 82 DF,  p-value: 0.5902
```

Adjusted regression models

Adjusted model with randomization

```
##
## Call:
## lm(formula = six_MWT_effect ~ rand_group + sex + sofa_score +
##     LOS_ICU_Insel_W + ADL_restr + Bed_EOB_OOB, data = icuaw)
##
## Residuals:
##      Min       1Q  Median      3Q     Max
## -260.36 -116.93  -16.14  119.66  389.00
##
## Coefficients:
##                 Estimate Std. Error t value Pr(>|t|)
## (Intercept)      284.712     54.255   5.248 1.42e-06 ***
## rand_groupyes     -1.932     36.657  -0.053   0.9581
## sexmale           58.737     35.756   1.643   0.1047
## sofa_score        -7.813      5.110  -1.529   0.1305
## LOS_ICU_Insel_W   -1.572      1.968  -0.799   0.4271
## ADL_restryes     -60.895     63.028  -0.966   0.3371
## Bed_EOB_OOB1    -111.496     63.310  -1.761   0.0824 .
## Bed_EOB_OOB2     -21.809     41.781  -0.522   0.6032
## ---
## Signif. codes:  0 '***' 0.001 '**' 0.01 '*' 0.05 '.' 0.1 ' ' 1
##
## Residual standard error: 153.5 on 74 degrees of freedom
##   (33 observations deleted due to missingness)
## Multiple R-squared:  0.1314, Adjusted R-squared:  0.0492
## F-statistic: 1.599 on 7 and 74 DF,  p-value: 0.1491
```

Adjusted model with randomization without ADL (activities of daily living)

```
##
## Call:
## lm(formula = six_MWT_effect ~ rand_group + sex + sofa_score +
##     LOS_ICU_Insel_W + Bed_EOB_OOB, data = icuaw)
```

```
##
## Residuals:
##     Min      1Q  Median      3Q     Max
## -258.42 -119.78  -26.52  116.43  391.19
##
## Coefficients:
##                 Estimate Std. Error t value Pr(>|t|)
## (Intercept)      286.102     53.363   5.361 8.36e-07 ***
## rand_groupyes     -7.883     36.238  -0.218   0.8284
## sexmale           53.372     35.253   1.514   0.1341
## sofa_score        -7.669      5.062  -1.515   0.1338
## LOS_ICU_Insel_W   -1.515      1.958  -0.774   0.4414
## Bed_EOB_OOB1    -109.667     62.958  -1.742   0.0855 .
## Bed_EOB_OOB2     -37.243     40.219  -0.926   0.3573
## ---
## Signif. codes:  0 '***' 0.001 '**' 0.01 '*' 0.05 '.' 0.1 ' ' 1
##
## Residual standard error: 152.8 on 77 degrees of freedom
##   (31 observations deleted due to missingness)
## Multiple R-squared:  0.1174, Adjusted R-squared:  0.04863
## F-statistic: 1.707 on 6 and 77 DF,  p-value: 0.1306
```

Robust adjusted model with randomization with ADL

```
##
## Call:
## lmRob(formula = six_MWT_effect ~ rand_group + sex + sofa_score +
##     LOS_ICU_Insel_W + ADL_restr + Bed_EOB_OOB, data = icuaw)
##
## Residuals:
##     Min      1Q  Median      3Q     Max
## -253.28 -113.07  -10.43  123.88  399.78
##
## Coefficients:
##                 Estimate Std. Error t value Pr(>|t|)
## (Intercept)      278.704     73.790   3.777 0.000318 ***
## rand_groupyes      2.790     51.110   0.055 0.956612
## sexmale           54.821     51.162   1.072 0.287418
## sofa_score        -8.045      7.869  -1.022 0.309933
## LOS_ICU_Insel_W   -1.324      2.738  -0.484 0.630012
## ADL_restryes     -59.664     84.641  -0.705 0.483080
## Bed_EOB_OOB1    -105.676     86.094  -1.227 0.223546
## Bed_EOB_OOB2     -16.743     57.859  -0.289 0.773104
## ---
## Signif. codes:  0 '***' 0.001 '**' 0.01 '*' 0.05 '.' 0.1 ' ' 1
##
## Residual standard error: 163.2 on 74 degrees of freedom
## Multiple R-Squared: 0.1042
##
## Test for Bias:
##             statistic p-value
## M-estimate      2.736  0.9498
## LS-estimate    -1.734  1.0000
```

```
## 33 observations deleted due to missingness
```

Adjusted model without randomization with ADL

```
##
## Call:
## lm(formula = six_MWT_effect ~ sex + sofa_score + LOS_ICU_Insel_W +
##     ADL_restr + Bed_EOB_OOB, data = icuaw)
##
## Residuals:
##     Min      1Q  Median      3Q     Max
## -259.62 -116.65  -15.18  118.53  389.56
##
## Coefficients:
##                 Estimate Std. Error t value Pr(>|t|)
## (Intercept)      283.609     49.729   5.703 2.21e-07 ***
## sexmale           59.057     35.003   1.687   0.0957 .
## sofa_score        -7.786      5.050  -1.542   0.1273
## LOS_ICU_Insel_W   -1.579      1.949  -0.810   0.4204
## ADL_restryes     -61.017     62.566  -0.975   0.3326
## Bed_EOB_OOB1    -111.529     62.885  -1.774   0.0802 .
## Bed_EOB_OOB2     -22.486     39.493  -0.569   0.5708
## ---
## Signif. codes:  0 '***' 0.001 '**' 0.01 '*' 0.05 '.' 0.1 ' ' 1
##
## Residual standard error: 152.5 on 75 degrees of freedom
##   (33 observations deleted due to missingness)
## Multiple R-squared:  0.1313, Adjusted R-squared:  0.06184
## F-statistic:  1.89 on 6 and 75 DF,  p-value: 0.09358
```

Robust adjusted model without randomization with ADL

```
##
## Call:
## lmRob(formula = six_MWT_effect ~ sex + sofa_score + LOS_ICU_Insel_W +
##     ADL_restr + Bed_EOB_OOB, data = icuaw)
##
## Residuals:
##      Min       1Q   Median       3Q      Max
## -257.488 -108.089   -9.784  123.728  402.237
##
## Coefficients:
##                 Estimate Std. Error t value Pr(>|t|)
## (Intercept)      284.225     59.873   4.747 9.67e-06 ***
## sexmale           57.126     41.669   1.371    0.174
## sofa_score        -9.227      6.602  -1.398    0.166
## LOS_ICU_Insel_W   -1.067      2.281  -0.468    0.641
## ADL_restryes     -57.552     75.166  -0.766    0.446
## Bed_EOB_OOB1    -102.903     71.399  -1.441    0.154
## Bed_EOB_OOB2     -16.743     46.256  -0.362    0.718
## ---
## Signif. codes:  0 '***' 0.001 '**' 0.01 '*' 0.05 '.' 0.1 ' ' 1
##
```

```
## Residual standard error: 159.2 on 75 degrees of freedom
## Multiple R-Squared: 0.09883
##
## Test for Bias:
##             statistic p-value
## M-estimate     1.1225  0.9926
## LS-estimate   -0.5153  1.0000
## 33 observations deleted due to missingness
```

Additive adjusted model without randomization with ADL

```
##
## Family: gaussian
## Link function: identity
##
## Formula:
## six_MWT_effect ~ sex + sofa_score + s(LOS_ICU_Insel_W) + ADL_restr +
##     Bed_EOB_OOB
##
## Parametric coefficients:
##              Estimate Std. Error t value Pr(>|t|)
## (Intercept)   268.560     50.256   5.344 9.43e-07 ***
## sexmale        59.057     35.003   1.687   0.0957 .
## sofa_score     -7.786      5.050  -1.542   0.1273
## ADL_restryes  -61.017     62.566  -0.975   0.3326
## Bed_EOB_OOB1 -111.529     62.885  -1.774   0.0802 .
## Bed_EOB_OOB2  -22.486     39.493  -0.569   0.5708
## ---
## Signif. codes:  0 '***' 0.001 '**' 0.01 '*' 0.05 '.' 0.1 ' ' 1
##
## Approximate significance of smooth terms:
##                     edf Ref.df     F p-value
## s(LOS_ICU_Insel_W)   1      1 0.656    0.42
##
## R-sq.(adj) =  0.0618   Deviance explained = 13.1%
## GCV =  25418  Scale est. = 23248     n = 82
```

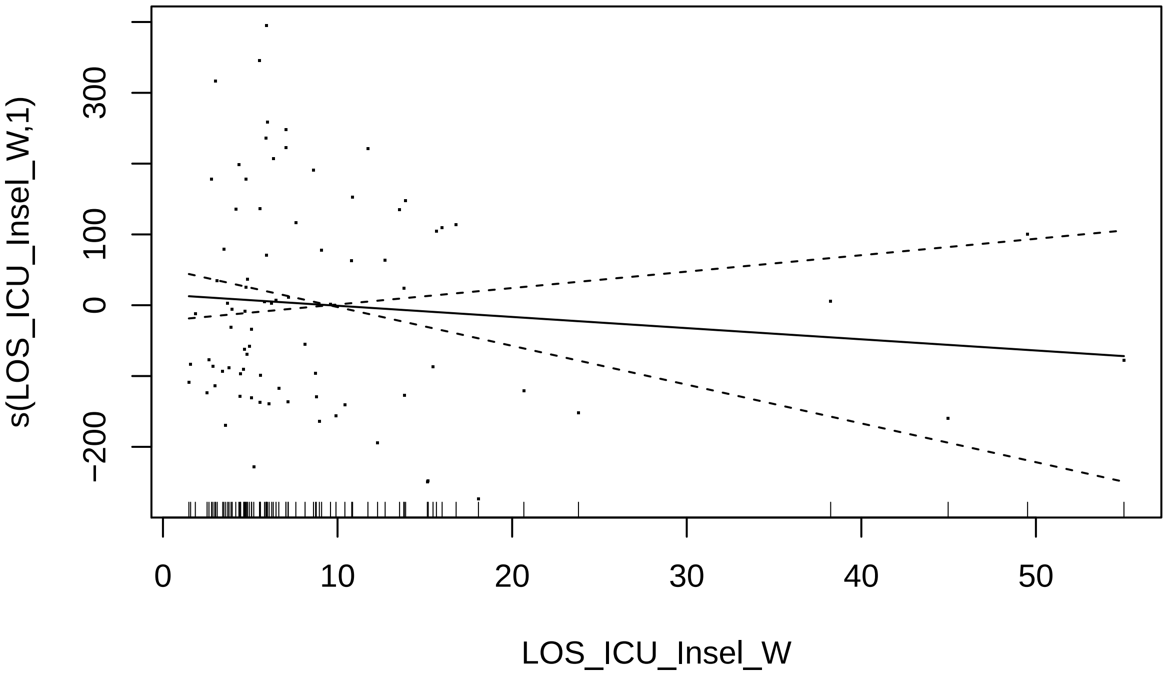

The edf is very close to 1 (1.4) and the plot shows rather a linear term, so the non-linear model is not worth using
